# Polymerization Shrinkage of Short Fiber Reinforced Dental Composite Using a Confocal Laser Analysis

**DOI:** 10.3390/polym13183088

**Published:** 2021-09-13

**Authors:** Daisuke Miura, Yoshiki Ishida, Akikazu Shinya

**Affiliations:** 1 Department of Dental Materials Science, School of Life Dentistry at Tokyo, The Nippon Dental University, 1-9-20, Fujimi, Chiyoda-ku, Tokyo 102-8159, Japan; daisuke@tky.ndu.ac.jp (D.M.); yishida@tky.ndu.ac.jp (Y.I.); 2 Turku Biomaterials Research Program, Department of Biomaterials Science, Institute of Dentistry and BioCity, University of Turku, Lemmikäisenkatu 2, 20520 Turku, Finland

**Keywords:** fiber reinforced composite, polymerization shrinkage stress, biomimetics restoration, bulk fill dental composite resin, short fiber reinforced dental composite resin, multicolor confocal displacement laser, dental materials, contraction gap, aspect ratio of glass fiber, dental composite resin

## Abstract

The purpose of this study was to investigate the polymerization shrinkage of short fiber reinforced composite (SFRC) using a multicolor confocal displacement laser that can measure the polymerization shrinkage with high accuracy. The three types of SFRCs used in this study were XD (Ever X Flow Dentin), XB (Ever X Flow Bulk), and XP (EverX Posterior). In addition, CF (Clearfil majesty ES Flow) with hybrid type filler was used as a control. The measured values of the final polymerization shrinkage rate and amount of polymerization shrinkage rate when the polymerization shrinkage rate became constant (less than 0.1 µm/s) were approximated for all SFRCs. XP had a large aspect ratio of glass fiber filler and showed a significant difference from XD with a small aspect ratio (*p* < 0.05). There was no significant difference in the measured value of time when the polymerization contraction reached a constant speed (0.1 µm/s or less) for all SFRCs (*p* > 0.05). There was no significant difference in the measured values of polymerization shrinkage rate after the polymerization shrinkage reached a constant rate for all SFRCs (*p* > 0.05). These results show that glass fiber with large aspect ratio can alleviate polymerization shrinkage stress. The polymerization behavior of SFRC was found to be dependent on the amount of glass fiber filler, aspect ratio, and orientation.

## 1. Introduction

Dental composite resins (CR) are widely used in restorative dentistry, because they allow us to restore teeth aesthetically while preserving healthy tooth substances using an adhesive system [1,2,3,4,5,6,7,8,9]. They also feature bonding systems that allow them to adhere firmly to the tooth structure and have a natural coloration. In recent years, the demand for composite resin has been expanding further due to the emergence of zirconia filler, nano-filler, or nano-hybrid filler, which improves the low durability of resin materials and realizes high wear resistance and high elastic modulus. However, polymerization shrinkage is an unavoidable problem in CR restorations. If this shrinkage forms a gap between the CR and cavity, it might cause secondary caries [3,10,11]. These problems are related to the low flexural strength of CRs and large polymerization shrinkage caused by the base monomer. CR undergoes volumetric shrinkage during polymerization, which is considered to be post-gelation shrinkage. This shrinkage ranges from 1 to 4.5% and is often one of the main causes of secondary caries and failure of bonded restorations [12,13]. Ethylene Glycol Dimethacrylate is mainly used as a polymer matrix for dental materials. Residual unreacted double bonds during curing can cause poor performance of the resin [14,15]. Therefore, the reaction rate of the double bond should be as high as possible, but the reaction rate will never reach 100%. Therefore, there has been no significant progress in improving the properties of the polymer matrix, and the recent improvements in the properties of CR are mainly due to fillers [16]. Changes in filler content have a significant effect on thermal expansion, thermal conductivity, polymerization shrinkage, and mechanical strength [17,18,19,20,21]. Compared with conventional CR, flowable composite resin has less polymerization shrinkage stress and therefore less gaps, but its instrumental properties are small. Therefore, these properties have been improved by varying the size of the filler or increasing the filling ratio of the granular filler [9,22,23]. Recently, short fiber reinforced composite (SFRC) has been developed for direct restorative and indirect prosthetic applications. It has been reported that the flexural properties of the SFRC can be significantly improved by filling randomly oriented short glass fibers into the base monomer. William R et al. also reported that the addition of silane-treated glass fibers to BIS-GMA resin improved instrumental properties. Paul S et al. reported that short glass fibers can be used in flowable dental composites to produce light-cured, short glass fiber-reinforced flowable materials with superior flexural properties compared to conventional CR. Lippo L et al. reported improved mechanical performance when used in a continuous fiber filler polymer matrix at different length scales compared to conventional CR [24,25,26]. However, although there are many reports on the mechanical properties of SFRCs, there are few studies on polymerization shrinkage The amount of glass fiber filling, fiber orientation, and aspect ratio in the SFRC are expected to affect the shrinkage of the CR during curing. Inadequate polymerization may lead to recurrence of caries, impact on pulp, or formation of gaps [27,28]. Therefore, proper measurement of polymerization shrinkage and clarification of the behavior of polymerization shrinkage are clinically important and have been the subject of various studies.

The measurement methods used so far include the mercury expansion measurement method, bonded disk method, strain gauge method, and shrinkage stress. Among them, measurement with a laser displacement meter is considered to be particularly accurate because it can measure the displacement of materials without contact, and no measurement load is applied [29]. However, the conventional semiconductor laser displacement transducer has only one type of light source, and when the object is transparent or specular, not enough reflected light reaches the light receiving part, making it difficult to obtain accurate measurement values [30]. The confocal laser displacement transducer has three light sources and measures only the light of the most focused wavelength at the time of receiving light. Thus, it can obtain accurate measurement values even for objects with high light transmittance, such as CRs occurring in the diffuse reflection of light due to the presence of fillers [31]. In this way, the confocal laser displacement meter can be applied to the measurement of dimensional accuracy of various materials, but there are few studies on its application to dental materials.

In this study, we aimed to investigate the polymerization behavior of three different SFRCs in detail using a confocal laser displacement meter.

## 2. Materials and Methods

**Figure 1:**
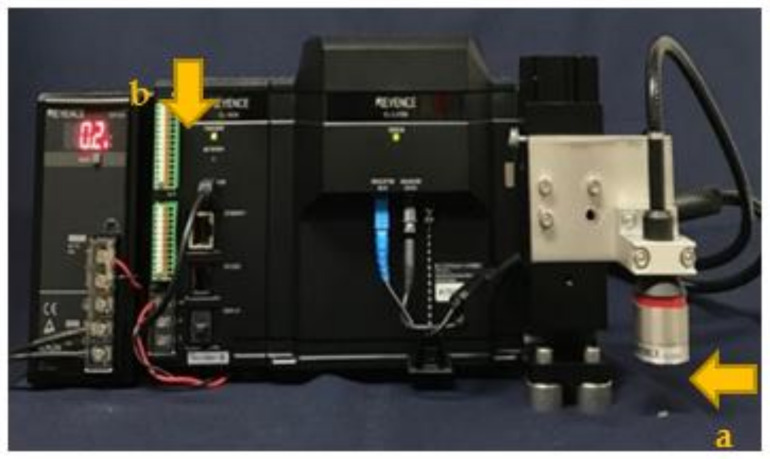
Multicolor confocal displacement laser used in this study for measuring the shrinkage of short fiber reinforced composites. (**a**): a center head CL-L007G, Keyence, Osaka, Japan, (**b**): optical unit CL-L015, Keyence, Osaka, Japan.

Table 1 shows the materials used in this study. Three types of SFRC and a flowable CR as a control were used. The laser displacement transducer was assembled by a multi-color confocal laser, which consists of a center head (CL-L015, Keyence, Osaka, Japan) and an optical unit (CL-L007G, Keyence, Osaka, Japan). The center head was mounted on a steel plate, and the laser head was fixed to a magnetic stand using a self-made mounting jig (Figure 1).

The settings of measuring equipment were as follows. Sampling period: 1000 ms, number of stored data: 5500, measurement interval: 15/60 s, measurement time: 90 min. A glass plate (thickness 5 mm × width 25 mm × length 10 mm) coated with a silane coupling agent (ceramic primer 2, GC, Tokyo, Japan) was applied to the steel plate (thickness 50 mm × width 250 mm × length 250 mm). Additionally, a glass tube (8 mm outer diameter, 5 mm inner diameter × 10 mm height) coated with a separator (New Acrozep, GC, Tokyo, Japan) was placed perpendicular to the glass plate. SFRC were filled to about 90% (approximately 0.5 g) of the height of the glass tube. A multicolor eutectic-point laser displacement meter was then fixed at a position of 30 mm from the upper surface of SFRC. The lower half of the SFRC was irradiated three times for 10 s each from a direction perpendicular to the long axis at an average intensity of 1000 mW/cm^2^ using a light irradiator or composite resin restoration (VALO curing light, ULTRADENT, South Jordan, UT, USA) from a position that divided the circumference of the glass tube into three equal parts. After that, the upper half of the SFRC was irradiated in the same way as the lower half, and a total of 60 s of light irradiation was performed.

The final polymerization shrinkage of short fiber reinforced composites was calculated from the total amount of polymerization shrinkage after 90 min. The polymerization shrinkage speed of short fiber reinforced composites was calculated from the linear regression of the polymerization shrinkage curve. The point at which the slope became less than 0.0005 was considered where the polymerization shrinkage was a plateau. From this point, the amount of polymerization shrinkage rate when the polymerization shrinkage becomes a constant speed of short fiber reinforced composites (PSC), the time when the polymerization shrinkage becomes a constant speed of short fiber reinforced composites (TPS), and the amount of polymerization shrinkage rate after the polymerization shrinkage becomes a constant speed of short fiber reinforced composites (PSA) were measured. The measurements were repeated five times (*n* = 5), and one-way analysis of variance was performed after confirming equal variance for each measurement. Paired comparisons by Tukey were performed for those with significant differences. All experiments were conducted by the same person.

## 3. Results

**Figure 2:**
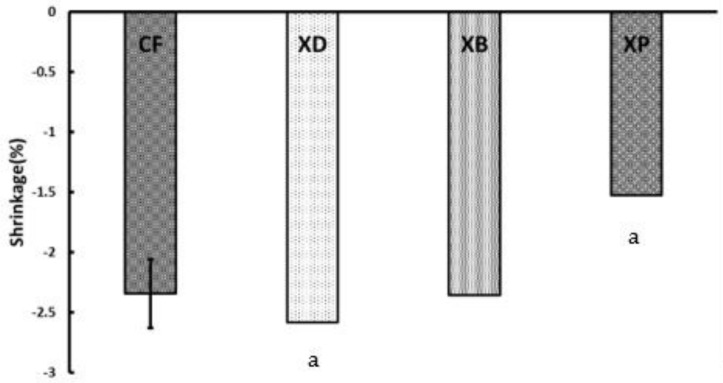
Final polymerization shrinkage rate of short fiber reinforced composites. (Significant difference between the same letters: *p* < 0.05, I: 95% confidence interval).

**Figure 3:**
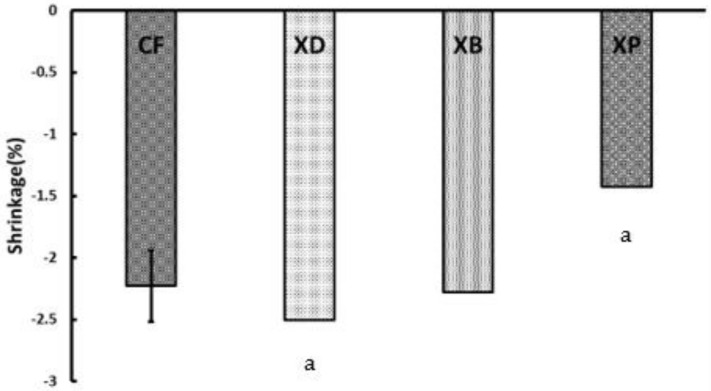
Polymerization shrinkage rate amount when the polymerization shrinkage speed becomes constant of short fiber reinforced composites (0.1 µm/s or less) (PSC). (Significant difference between the same letters: *p* < 0.05, I: 95% confidence interval).

Analysis of variance was performed on the measured values of final polymerization shrinkage rate and PSC. As a result, a significant difference (*p* < 0.05) was found among the samples, and the results are shown in Figure 2 and Figure 3.

In the final polymerization shrinkage rate, XP showed the smallest value of −1.52%, and XD showed the largest value of −2.58%, indicating a significant difference between XP and XD (*p* < 0.05). In the same way, XP showed the smallest value of −1.42%, and XD showed the largest value of −2.50% in PSC, indicating a significant difference between XP and XD (*p* < 0.05). The final amount of polymerization shrinkage for CF (−2.34%) was comparable to that of XD and XB (−2.35%), and no significant difference was observed. In PSC, XD, and XB no significant difference was observed.

**Figure 4:**
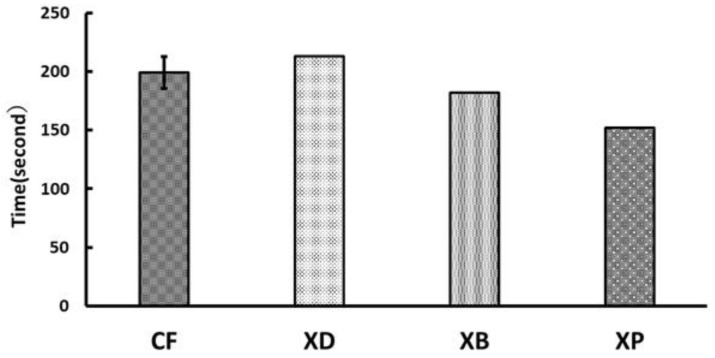
Time when the polymerization contraction reached a constant speed of short fiber reinforced composites (0.1 µm/s or less) (TPS) (I: 95% confidence interval).

**Figure 5:**
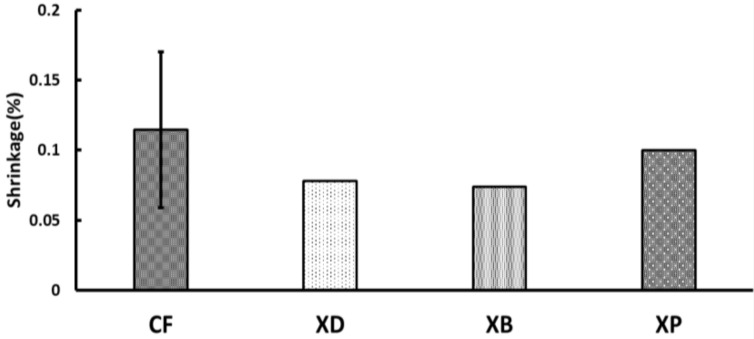
Amount of polymerization shrinkage rate after polymerization shrinkage reaches a constant speed of short fiber reinforced composites (0.1 µm/s or less) (PSA) (I: 95% confidence interval).

A graph of TPS is shown in Figure 4. There was no significant difference among all the materials (*p* > 0.05), and the speed became constant almost within 200 s, with XP being the fastest at 152.2 s and XD being the slowest at 212.8 s. The graph of the measured values of PSA is shown in Figure 5. There was no significant difference among all samples, with XB being the smallest (0.078%) and XP being the largest (0.10%), comparable to CF.

**Figure 6:**
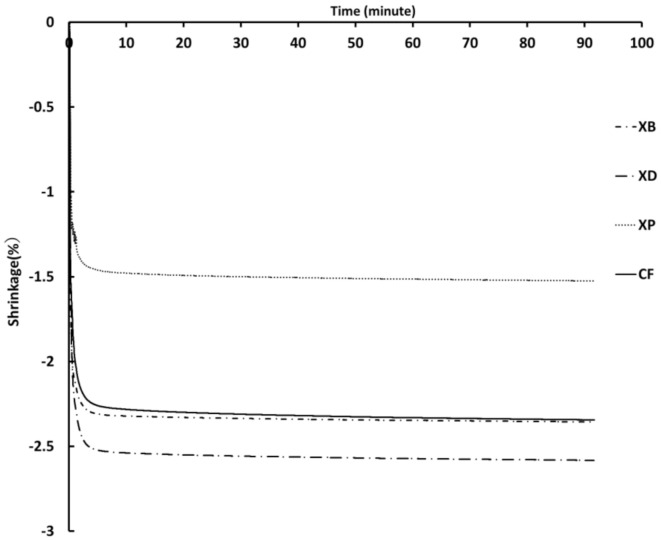
Curves of the polymerization shrinkage on short fiber reinforced composites.

The polymerization shrinkage curves are shown in Figure 6. In all materials, a rapid volume change was observed in the 5 min measurement after light irradiation. After that, a gradual shrinkage curve was observed after 10 min of light irradiation. CF and XB showed similar shrinkage curves, and the final polymerization shrinkage of XP was approximately 1% smaller than that of XD.

## 4. Discussion

Photopolymerized resins undergo polymerization shrinkage due to the shortening of the intermolecular distance between monomers during polymerization and curing. This polymerization shrinkage forms a contraction gap, which causes postoperative pain and marginal leakage [32,33].

Therefore, accurate measurement of the polymerization shrinkage behavior of resin is an important factor for CR restoration. The laser displacement transducer is suitable for the measurement of dimensional changes due to the detailed polymerization shrinkage behavior because it can measure the mutation of the material without contact and the measurement load is zero. Conventional laser displacement transducers measure distances by irradiating a laser beam on an object and concentrating the reflected light on a light-receiving lens, so that the measured value is affected by the surface properties of the object [29].

This makes it difficult to make accurate measurements for materials with rough surfaces, multiple laser reflections, and light transmission.

In this study, the contraction behavior of SFRC was compared using a multicolor confocal laser displacement meter, which is capable of accurate measurement.

The total filler content of XP (65–85%) is higher than that of XD (67–77%) and XB (67–77%), but the fiber content of XP (5–15%) is lower than that of XD (25%) and XB (25%). In the final polymerization shrinkage rate, XP (−1.52%) showed the smallest value. This result may be due to the fiber size of XP and the behavior of fiber content. SFRC is known to have a stress relaxation function, relieve polymerization shrinkage stress, and reduce polymerization shrinkage (9). The aspect ratio, amount of content, and orientation of the fiber are thought to be involved in this stress relaxation. The fiber of XP (17 µm in diameter × 800 µm in length) has a larger aspect ratio than that of XD and XB (6 µm in diameter × 140 µm in length). Therefore, when filled into the glass tube, the fiber orientation will be remaining parallel in the center of the resin, and the fibers will intertwine with each other in the inner wall of the glass tube and in the filled area. In addition, the polymerization shrinkage along the glass fiber fibers is considered to be limited [34,35]. This indicates that the matrix resin between the fibers polymerized and shrank, but the intertwined fiber absorbed the stress at the inner wall of the glass tube where the most polymerization shrinkage stress was applied. As a result, the final polymerization shrinkage rate of XP is considered to be the smallest value. XP also showed the smallest values for TPS and PSA. These results are also partly due to the fact that the fibers with large aspect ratios in the XP intertwined with each other, which alleviated the polymerization shrinkage stress.

The polymerization shrinkage curve in Figure 6 also shows the difference in polymerization shrinkage stress relaxation depending on the aspect ratio of the fiber. All SFRCs show rapid volume shrinkage within 5 min of light irradiation. Among them, XP shows a slow polymerization shrinkage curve about one minute earlier than CF, XD, and XB. Therefore, by filling the cavity with SFRC with a large aspect ratio, the fibers intertwine with each other at the wall surface, which alleviates the polymerization shrinkage stress caused by the rapid volume change during light irradiation. As a result, it can be said that particularly effective against the occurrence of white margin and contrast gap.

The morphology of the filler in the CR is a factor that affects the polymerization depth, and fiber is known to propagate and scatter light more than conventional spherical filler [36,37,38,39,40,41]. The amount of fiber in XD and XB is about twice that of XP, and because of their small aspect ratio, they are considered to be evenly dispersed in the glass tube without tangling. Then, it is suggested that the polymerization reaction remaining inside the CR after light irradiation is dispersed by with a small aspect ratio, and the polymerization shrinkage stress is relaxed. As a result, the PSA of XD and XB is considered to be smaller than that of CF and XP.

From these results, it was found that there was a difference in the shrinkage behavior of SFRC depending on the aspect ratio and the amount of fiber as a filler. Fibers with a large aspect ratio intertwine with each other in the cavity and are effective in alleviating the large polymerization shrinkage stress during light irradiation. The fiber with a small aspect ratio can conduct and scatter the irradiated light to every corner of the cavity. Therefore, it was effective in alleviating the polymerization shrinkage stress after light irradiation. This behavior is thought to be important in deep cavities where it is difficult to be irradiated. In this study, we found that the polymerization shrinkage behavior differs depending on the aspect ratio, the amount of fiber, and the orientation of the filler used by the multicolor confocal displacement laser.

High polymerization shrinkage stress causes gaps, such as contrast gaps and white margins in the cavity and bonded surfaces. Bacteria can enter through these gaps and cause caries and pulpitis. In addition, staining of the margins occurs due to the penetration of dyes [42,43,44,45,46,47,48].

The ability of SFRCs to relax polymerization shrinkage stresses may be a particularly important behavior for white margins and contrast gaps that occur during direct restorations.

In this study, simple cylindrical samples were used for the measurement. However, in daily clinical practice, the cavities prepared have a more complex shape than those of this study, so the results obtained in this study might differ from the values measured in the actual cavity.

Therefore, further studies are needed to clarify it.

## 5. Conclusions

In this study, using a multicolor confocal laser displacement meter, it was found that the polymerization behavior of SFRCs depended on the aspect ratio, the amount of glass fiber filler, and the orientation of the glass fiber filler.

SFRCs with a large fiber aspect ratio were found to relax the rapid polymerization shrinkage stress induced by light irradiation. SFCR with a small aspect ratio was found to alleviate the polymerization shrinkage stress after light irradiation.

Glass fiber filler was found to be importantly related to the transfer of light irradiation energy used in composite resin restorations. The aspect ratio of the glass fiber filler was also considered to be an important factor, but the morphology and dimensions of the cavity may also depend on these results. In future studies, it is necessary to investigate how the polymerization behavior of SFRC changes depending on the dimensions of the cavity.

It was suggested that the glass fiber affected the transmission of light irradiation energy and was effective in suppressing secondary caries and white margin.

## Figures and Tables

**Table 1:** Composite resins used in this study.

Code	Product Name	Base Monomer	Filler	Filler Content (wt%)	Shade	Manufacture
CF	Clearfil majesty ES Flow	Bis-GMA	Hybrid	78	A2	Kuraray Noritake Dental, Tokyo, Japan
XD	Ever X Flow (Dentin)	Bis-MEPP TEGDMA UDMA	Glass fibres Barium glass Silicon dioxide	25 42–52 Trace	Dentin	GC, Tokyo, Japan
XB	Ever X Flow (Bulk)	Bis-MEPP TEGDMA UDMA	Glass fibres Barium glass Silicon dioxide	25 42–52 Trace	Bulk Shade	GC, Tokyo, Japan
XP	EverX Posterior	Bis-GMA TEGDMA	Glass fibres Barium glass Silicon dioxide	5–15 60–70 1–5	Universal	GC, Tokyo, Japan

Bis-GMA: Bisphenol A glycidyl meth-acrylate; Bis-MEPP: Bisphenol a ethoxylate dimethacrylate; TEGDMA: Triethylene glycol di-methacrylate; UDMA: Diurethane dimethacrylate.

## Data Availability

Data of the study are available on request form the corresponding author.
